# Disruption of the *foxe1* gene in zebrafish reveals conserved functions in development of the craniofacial skeleton and the thyroid

**DOI:** 10.3389/fcell.2023.1143844

**Published:** 2023-03-13

**Authors:** Sophie T. Raterman, Johannes W. Von Den Hoff, Sietske Dijkstra, Cheyenne De Vriend, Tim Te Morsche, Sanne Broekman, Jan Zethof, Erik De Vrieze, Frank A. D. T. G. Wagener, Juriaan R. Metz

**Affiliations:** ^1^ Department of Dentistry—Orthodontics and Craniofacial Biology, Radboud Institute of Molecular Life Sciences (RIMLS), Radboud University Medical Center, Nijmegen, Netherlands; ^2^ Department of Animal Ecology and Physiology, Radboud Institute for Biological and Environmental Sciences (RIBES), Radboud University, Nijmegen, Netherlands; ^3^ Department of Human Genetics, Radboud University Medical Center, Nijmegen, Netherlands

**Keywords:** zebrafish, FOXE1, bamforth-lazarus syndrome, cleft palate, craniofacial malformations, skeletal development, thyroidogenesis

## Abstract

**Introduction:** Mutations in the FOXE1 gene are implicated in cleft palate and thyroid dysgenesis in humans.

**Methods:** To investigate whether zebrafish could provide meaningful insights into the etiology of developmental defects in humans related to FOXE1, we generated a zebrafish mutant that has a disruption in the nuclear localization signal in the foxe1 gene, thereby restraining nuclear access of the transcription factor. We characterized skeletal development and thyroidogenesis in these mutants, focusing on embryonic and larval stages.

**Results:** Mutant larvae showed aberrant skeletal phenotypes in the ceratohyal cartilage and had reduced whole body levels of Ca, Mg and P, indicating a critical role for foxe1 in early skeletal development. Markers of bone and cartilage (precursor) cells were differentially expressed in mutants in post-migratory cranial neural crest cells in the pharyngeal arch at 1 dpf, at induction of chondrogenesis at 3 dpf and at the start of endochondral bone formation at 6 dpf. Foxe1 protein was detected in differentiated thyroid follicles, suggesting a role for the transcription factor in thyroidogenesis, but thyroid follicle morphology or differentiation were unaffected in mutants.

**Discussion:** Taken together, our findings highlight the conserved role of Foxe1 in skeletal development and thyroidogenesis, and show differential signaling of osteogenic and chondrogenic genes related to foxe1 mutation.

## Introduction

Disruption of sonic hedgehog signaling during development can result in varying craniofacial malformations, as observed in clinical examples and confirmed in animal models (Wada et al., 2005; Xavier et al., 2016). Forkhead box transcription factor E 1 (FOXE1, also known as TTF-2) is a transcriptional regulator associated with the SHH pathway, that uses a forkhead DNA-binding domain to regulate transcription of downstream targets (Clifton-Bligh et al., 1998; Brancaccio et al., 2004; Eichberger et al., 2004). FOXE1 can be activated by GLI2 and regulates the transcription of genes involved in craniofacial development, including a transcription factor (*MSX1*) and growth factors (*TGFβ3, WNT5A*) and regulates thyroid differentiation factors (*TPO, NIS, TG*) (Venza et al., 2011; Lopez-Marquez et al., 2019).

**FIGURE 1:**
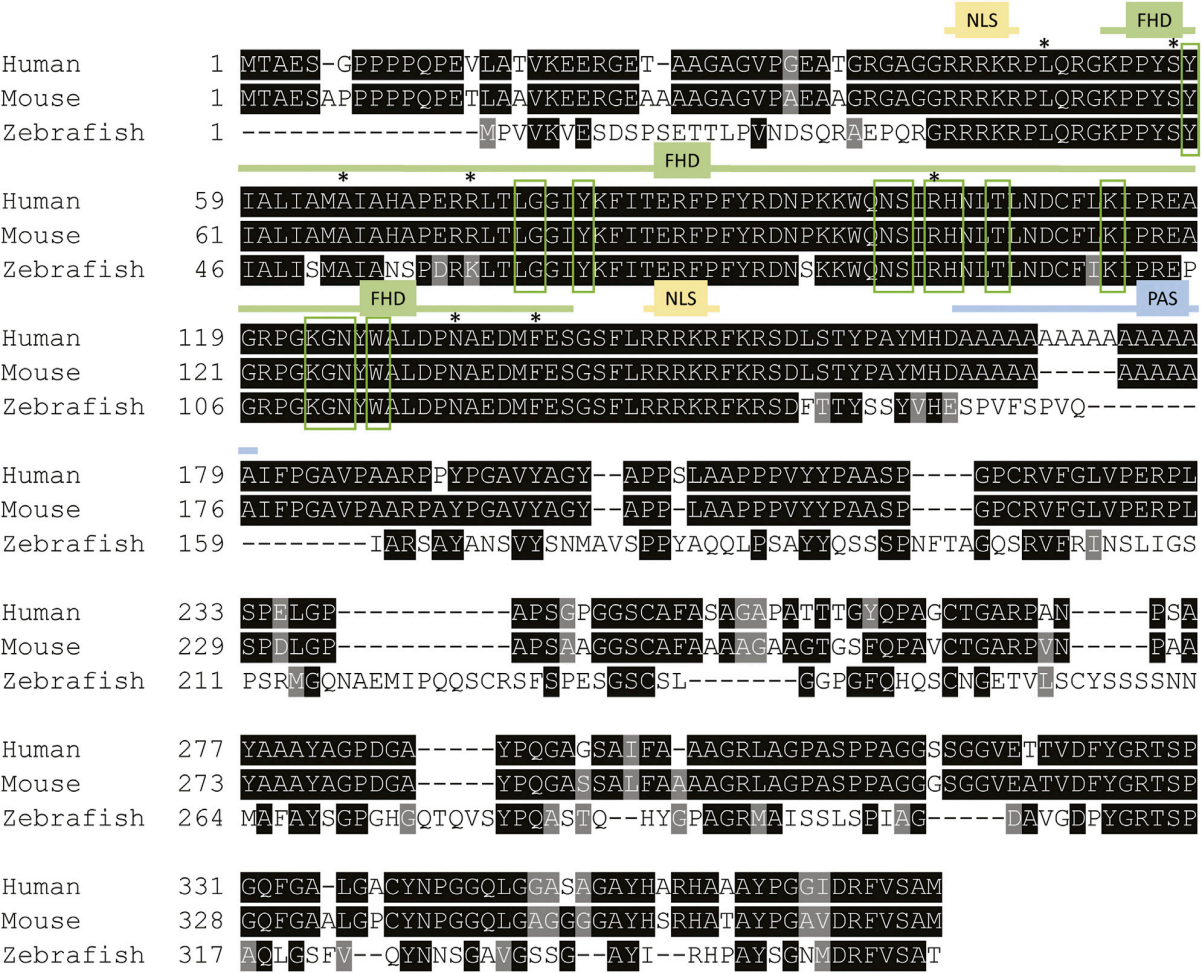
Foxe1 protein structure in human, mouse and zebrafish. Multiple sequence alignment of full-length human, mouse and zebrafish FOXE1 protein. The protein contains three characterized domains which are annotated with colored lines. The similarity of the Forkhead domain (FHD) is 97% and 100%, respectively, between zebrafish and humans and between mouse and human. The sequence similarity of the whole protein between human and zebrafish is 54%. NLS is 100% identical between all species and the poly alanine stretch (PAS) is only present in humans and mouse. Red boxes mark the putative DNA-binding residues and asterisks (*) mark locations of missense mutations that were reported in individuals with Bamforth–Lazarus syndrome (Clifton-Bligh et al., 1998; Castanet et al., 2002; Baris et al., 2006; Castanet and Polak 2010; Carre et al., 2014; Sarma et al., 2022). NLS: nuclear localization signal, FHD: forkhead box domain PAS: poly alanine stretch in human and mouse FOXE1. Alignment made using Clustal Omega.

FOX-family proteins and their characteristic DNA-binding domains are highly conserved among vertebrates (Figure 1). *FOXE1* is a single exon gene containing two functional regions; the forkhead DNA-binding domain and a 16–19 residue polyalanine stretch (Zannini et al., 1996). Cytoplasmic FOXE1 gains entry to the nucleus by two identical FHD-flanking nuclear localization signals. These localization signals facilitate nuclear entry of FOXE1 by binding to importin-α upon which it is transported by importin-β (McLane and Corbett 2009). FOXE1 is also retained in the nucleus by NLS activity, thus the NLSs are paramount for transcriptional regulation by FOXE1 (Romanelli et al., 2003; Castanet and Polak 2010).

Homozygous loss- and gain-of-function missense mutations in the forkhead domain of *FOXE1* cause Bamforth–Lazarus syndrome, which is characterized by thyroid dysgenesis, cleft palate, spiky hair, as well as, in some cases, choanal atresia and bifid epiglottis (Bamforth et al., 1989; Castanet and Polak 2010; Carre et al., 2014). Very recently, a novel homozygous protein-truncating frameshift mutation in *FOXE1* outside the FHD (Leu29Profs*75) was also reported to cause Bamforth–Lazarus syndrome (Sarma et al., 2022). Bamforth–Lazarus syndrome is an extremely rare condition with an incidence of <1:1.000.000. However, variants in the *FOXE1* locus have also been shown to contribute significantly to non-syndromic orofacial clefts in multiple case-control and GWAS studies. Indeed, the gene is thought to contribute substantially to both CP and CLP cases (Moreno et al., 2009; Dixon et al., 2011; Lammer et al., 2016; Leslie et al., 2017).

How FOXE1 is involved in palate formation is largely unknown, and etiological mechanisms for FOXE1-related orofacial clefts remain therefore elusive. This prompts the need for research models to investigate genotype-phenotype associations. *Foxe1* ^−/−^ mice feature severe cleft palate and the migration of the thyroid primordium is blocked in these mutants, resulting in thyroid dysgenesis (De Felice et al., 1998). Indeed, these phenotypes partly recapitulate the symptoms of Bamforth-Lazarus syndrome, yet *Foxe1* ^−/−^ mice die upon birth due to the extent of the clefts. This limits the use of this model for post-embryonic research and there is thus a need for alternative models that can provide insight into the role of FOXE1 in pathogenesis.

Zebrafish allow the modeling of human congenital craniofacial disorders since developmental processes are well-conserved during evolution (Swartz et al., 2011; Mork and Crump 2015). Cartilage and bone elements of zebrafish and other vertebrates’ craniofacial skeleton originate primarily from cranial neural crest cells. Induction in the neural plate, epithelial-mesenchymal transition, and subsequent migration of CNCC to the pharyngeal arches of this vertebrate-specific cell population occurs in zebrafish within the first 24 h after fertilization (Schilling and Kimmel 1994; Kague et al., 2012). The craniofacial cartilages subsequently develop from the pharyngeal arches, and then function as a template for bone development. In vertebrate skeletal development, three modes of bone formation are described, which all occur in zebrafish. First, endochondral ossification describes the formation of bone from within a cartilage template, starting at a primary ossification center in the cartilage structure. Second, intramembranous or dermal ossification describes direct bone formation without a cartilage template. Third, perichondral ossification refers to the covering of a cartilage template with bone in a sheath-like manner (Tonelli et al., 2020; Valenti et al., 2020; Dietrich et al., 2021).

A zebrafish study using morpholino-mediated knock-down of *foxe1* reported defects in chondrogenesis, specifically shortening of the Meckel’s cartilage, and inverted or shortened ceratohyal cartilages (Nakada et al., 2009). In the same study, *fgfr2* upregulation was indicated as the main mediator of the detrimental effects on the cranial skeleton. In addition, the role of *foxe1* in thyroid development in zebrafish was questioned, as no differences in the expression of thyroid markers were observed upon knock-down of *foxe1* (Nakada et al., 2009). Today, stable mutant lines are considered in most instances superior over morphants, due to non-specific binding artifacts and p53-mediated apoptosis in morphants (Schulte-Merker and Stainier 2014; Kok et al., 2015; Duncan et al., 2017; El-Brolosy and Stainier 2017). Importantly, morpholinos induce transient knock-downs only, of which effects wear off before the onset of mineralization, while stable mutant lines allow the study of effects during the entire lifespan.

To investigate whether a zebrafish *foxe1* mutant model could provide meaningful insights into human developmental defects related to FOXE1, the present study aimed to characterize the function of Foxe1 in craniofacial development and thyroidogenesis in zebrafish. We generated a *foxe1* mutant and observed skeletal mineralization defects and CNCC dysregulation. We also found that the expression of genes involved in osteogenesis and chondrogenesis in early development are modulated by *foxe1* disruption.

## Results

### Foxe1 is expressed in multiple larval tissues, including the oral epithelium, ethmoid plate, and the thyroid follicles

**FIGURE 2:**
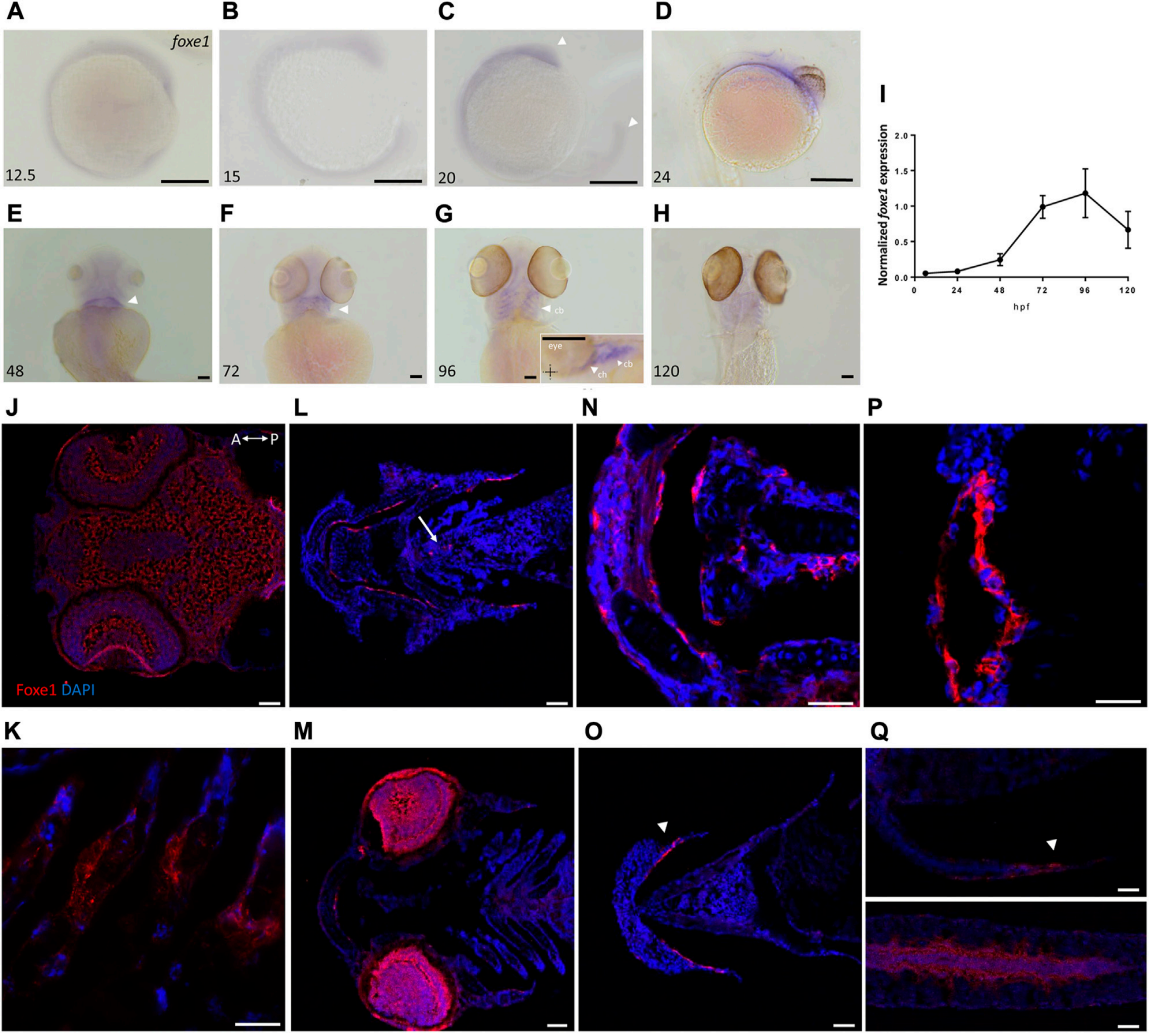
Foxe1 is expressed in a range of larval tissues, including the oral epithelium, ethmoid plate, and the brain. **(A–H)** *foxe1* transcripts were detected diffusely throughout the embryo from early development at 12.5 hpf **(A)** which continued through early larval stages **(B–D)** where transcripts also seemed specifically upregulated in the subpharyngeal area **(E, F)** before being enhanced in the ceratobranchials and ceratohyal (**(G)**, insert lateral view). **(A-D)** lateral view and **(E-H)** ventral view. **(I)** Normalized relative expression of *foxe1* during embryonic and larval stages as determined by qPCR. Error bars indicate standard deviations. **(J-Q)** Detailed protein localization at 96 hpf showed Foxe1-positive cells in the brain **(J)**, the ceratobranchials **(K)**, in the oral epithelium and in a linear cluster in the subpharyngeal area (white arrow) **(L)**, the eyes **(M)**, on the ethmoid plate **(N)**, on the lining of the ceratohyal **(O)**, in the mouth opening **(P)** and to some extent in the fin tips **(Q)**, upper image) and in the notochordal sheath (**(Q)**, lower image). Posterior-anterior axis in J applies to all images. cb: ceratobranchals, ch: ceratohyal. A-H scalebar 100 μm, **(J-Q)** scalebar 50 µm. Antibody validation in Supplementary Figure S1.

We first assessed spatiotemporal *foxe1* expression at both the transcriptional and the translational level in wild type zebrafish. *Foxe1* was diffusely detected by *in situ* hybridization at 12.5 hpf (Figure 2A) until 24 hpf in the head and notochord (Figures 2B–D, arrowheads in Figure 2C). A diffuse *foxe1* expression pattern was observed until 48 hpf with increased intensity in the thyroid primordium (Figure 2E); after that, *foxe1* continued to be expressed throughout the larvae and was specifically intense in the (sub)pharyngeal area (Figure 2F). At 96 hpf, *foxe1* expression was most abundant and was also observed in the cartilages of the viscerocranium (Figures 2G, I). Contrary to previous reports of *foxe1* spatiotemporal expression in the developing zebrafish larvae (Nakada et al., 2009; Lidral et al., 2015), in the present study *foxe1* expression was observed in the viscerocranial structures at 120 hpf. However, this expression was lower than that at 96 hpf (Figures 2H, I). Normalized gene expression levels reflected the patterns of *foxe1* expression throughout early development and indeed showed a sharp increase between 48 and 72 hpf as thyroid morphogenesis and craniofacial cartilage formation commenced (Figure 2I).

*Foxe1* was most abundantly expressed at 96 hpf, and a more detailed localization of the protein at this timepoint was examined by immunohistochemistry. Throughout the head we found Foxe1-positive cells, many of which would have been too scattered to be distinguished by whole mount *in situ* hybridization. Foxe1 was localized in the brain (Figure 2J), ceratobranchials (Figure 2K), oral epithelium (Figure 2L), eyes (Figure 2M), ethmoid plate (Figure 2N), lining of the ceratohyal cartilage (Figure 2O), mouth opening (Figure 2P), and, to some extent, in the tips of the fins and the notochordal sheath (Figure 2Q).

**FIGURE 3:**
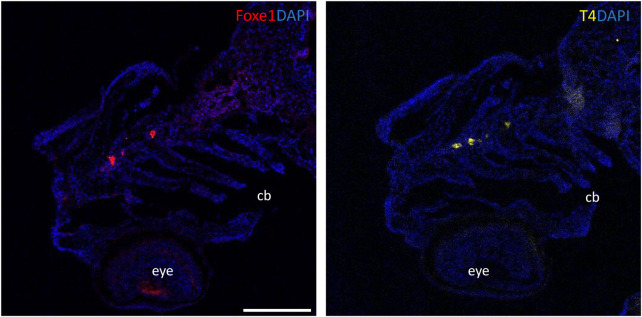
Foxe1 and T4 are colocalized in the subpharyngeal area. Foxe1 and T4 staining on adjacent coronal sections of the subpharyngeal area of the larval head. T4 and Foxe1 staining in the developing thyroid follicles at 96 hpf. Nuclear counterstain with DAPI. cb; ceratobranchials, e; eye. Scale bar 200 µm.

Foxe1-positive cells were also consistently observed in linear clusters in the subpharyngeal area at 96 hpf (indicated by the white arrow in Figure 2L). As Foxe1 is important in thyroid development in many vertebrates, we set out to establish whether this signal originated from the developing thyroid follicles. The zebrafish thyroid is not organized as a discrete gland, but as individual follicles scattered along the ventral aorta (Alt et al., 2006). These follicles appear from 72 to 96 hpf and can be visualized using thyroid hormone (T4) antibody staining (Zhang et al., 2021). Figure 3 shows two sequential sections of the same individual stained with Foxe1-and T4-antibodies. Thyroid hormone was observed in the colloid lumen of the thyroid follicle, where it is produced from its precursor thyroglobulin. Colocalization of the T4 signal with Foxe1 on follicular level was clearly detected and strongly supports a role for Foxe1 in the developing thyroid.

### *Foxe1* mutants show enhanced Foxe1 expression, normal growth rates, and normal thyroid follicle morphology

**FIGURE 4:**
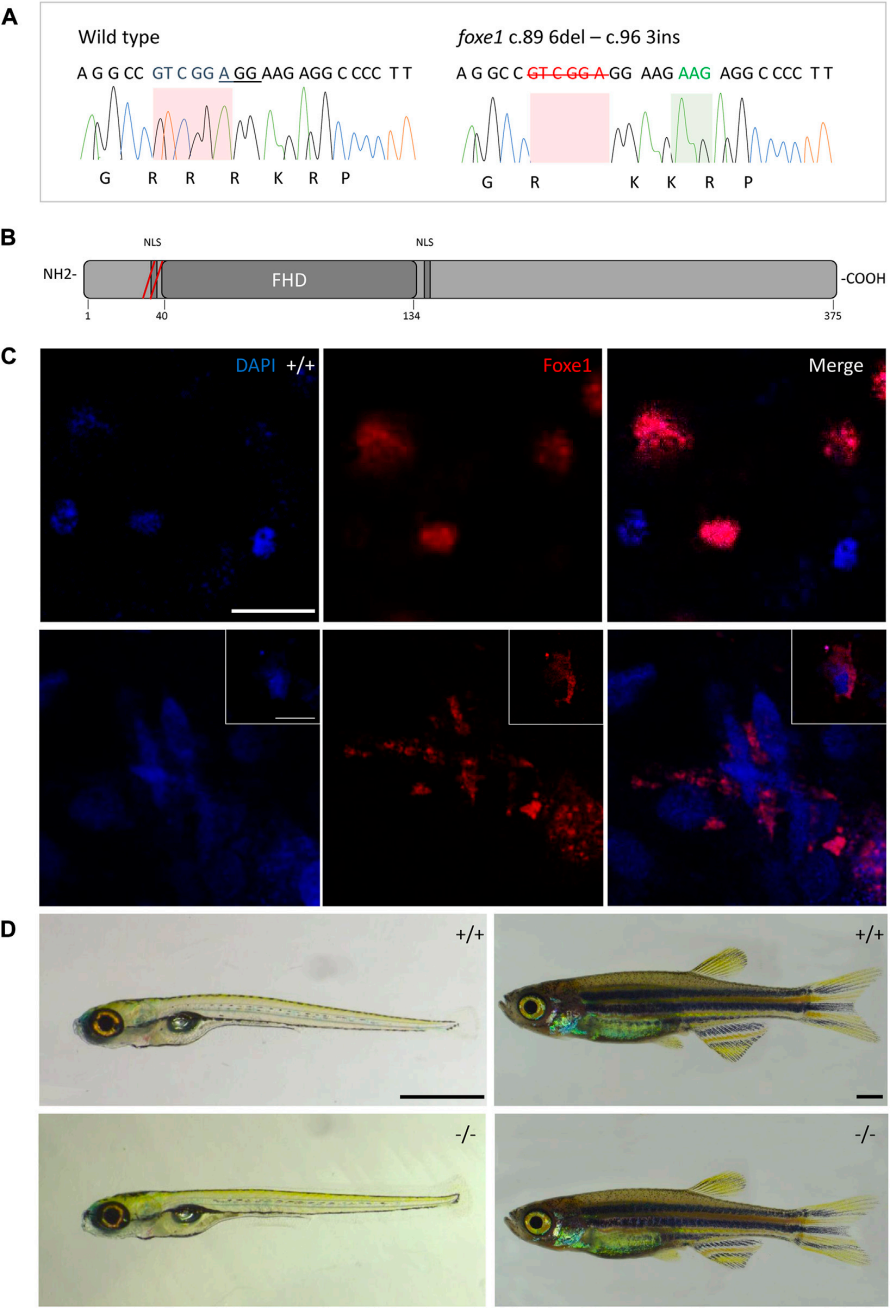
The *foxe1* ^rdb2^ mutant. **(A)** Wild type and *foxe1* ^rdb2^ mutant DNA sequence at **(C)** 84–105. The protospacer adjacent motif is underlined (full gRNA sequence: 5′-GCC​GCA​AAG​AGG​CCG​TCG​GAG​G-3′). The deletion is highlighted in red and the insertion in green. **(B)** Schematic representation of the zebrafish Foxe1 protein and the position of the amino acid changes as indicated by the red bars. **(C)** Nuclear localization of wild type Foxe1 and cytoplasmatic localization of mutant Foxe1 in zebrafish keratinocytes on scale. Scale bar 50 µm. **(D)** Representative images of wild types and *foxe1* mutants at 6 dpf and 2 months post fertilization. Scale bars 1 mm.

Although the role of Foxe1 in zebrafish thyroid follicle development was previously questioned, we here show that Foxe1 is expressed in the developing follicles (Nakada et al., 2009). To better understand the role of Foxe1 in craniofacial development and thyroidogenesis, we generated a *foxe1* mutant zebrafish line, using CRISPR-Cas9 targeting 5’ end of the single *foxe1* exon. Whilst screening the F1 generation, in-frame indels were identified with a 6-bp deletion and 3-bp insertion that we outcrossed to obtain a stable mutant line (which we termed *foxe1*^rdb2). These indels lead to an arginine deletion and arginine to leucine substitution in the NLS domain, resulting in the following amino acid sequence change: “RRRKR” to “RKKR” (Figures 4A, B). According to 3D structural predictions by homology modeling of the winged helix domain of Foxe1 in YASARA, this amino acid change alters the structure and function of the NLS domain in this mutant, affecting nuclear translocation and retention of Foxe1 (Supplementary Figure S2). Indeed, subcellular localization of Foxe1 shows that in wild types the protein is present inside the cell nucleus, while in mutants the disrupted NLS failed to translocate and retain Foxe1 into the nucleus and it was found to be localized mostly in the cytoplasm (Figure 4C).

**FIGURE 5:**
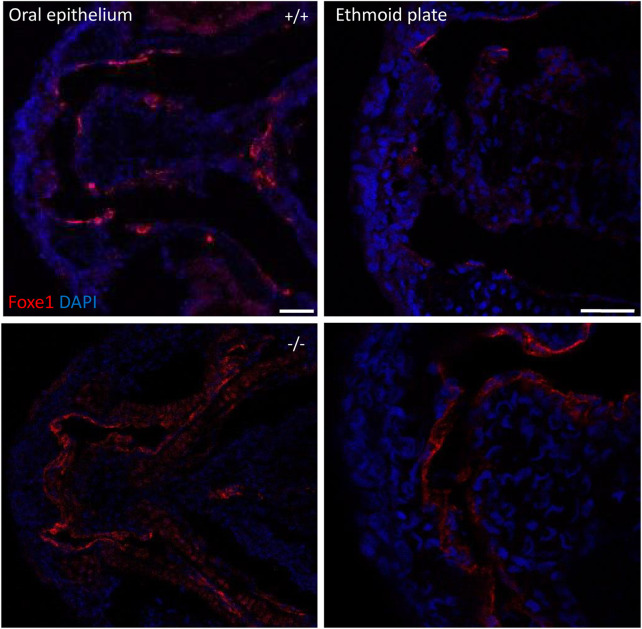
*foxe1* mutants show upregulated Foxe1 expression. Foxe1 expression in the oral epithelium and the ethmoid plate of wild type and *foxe1* mutants at 96 hpf. Foxe1 was more abundantly expressed in the mutants compared to the wild types, but the tissue localization of the protein remained unaffected. Scale bar 50 µm.

To assess the overall fitness of the homozygous *foxe1* ^rdb2^ mutants (referred to as mutant or −/− in the rest of the manuscript), survival, general morphology and growth rate analyses up to juvenile stages were performed in the F3 generation, according to previously established protocols (Parichy et al., 2009; Beekhuijzen et al., 2015). General morphology analyses included tracking of tail formation, pericardial development, and swim bladder formation. No significant differences between wild type, heterozygote, and homozygous mutant fish were observed in growth, development or survival (data not shown). Both the heterozygous and homozygous mutants were viable up to adulthood and fertile (Figure 4D). The immuno-localization of Foxe1 in different tissues was not altered by the in-frame mutation, but a more widespread abundance in the mutants was found (Figure 5). Overexpression of a mutated allele is a common feature of genetic mutants and can indicate loss-of-function (El-Brolosy et al., 2019).

**FIGURE 6:**
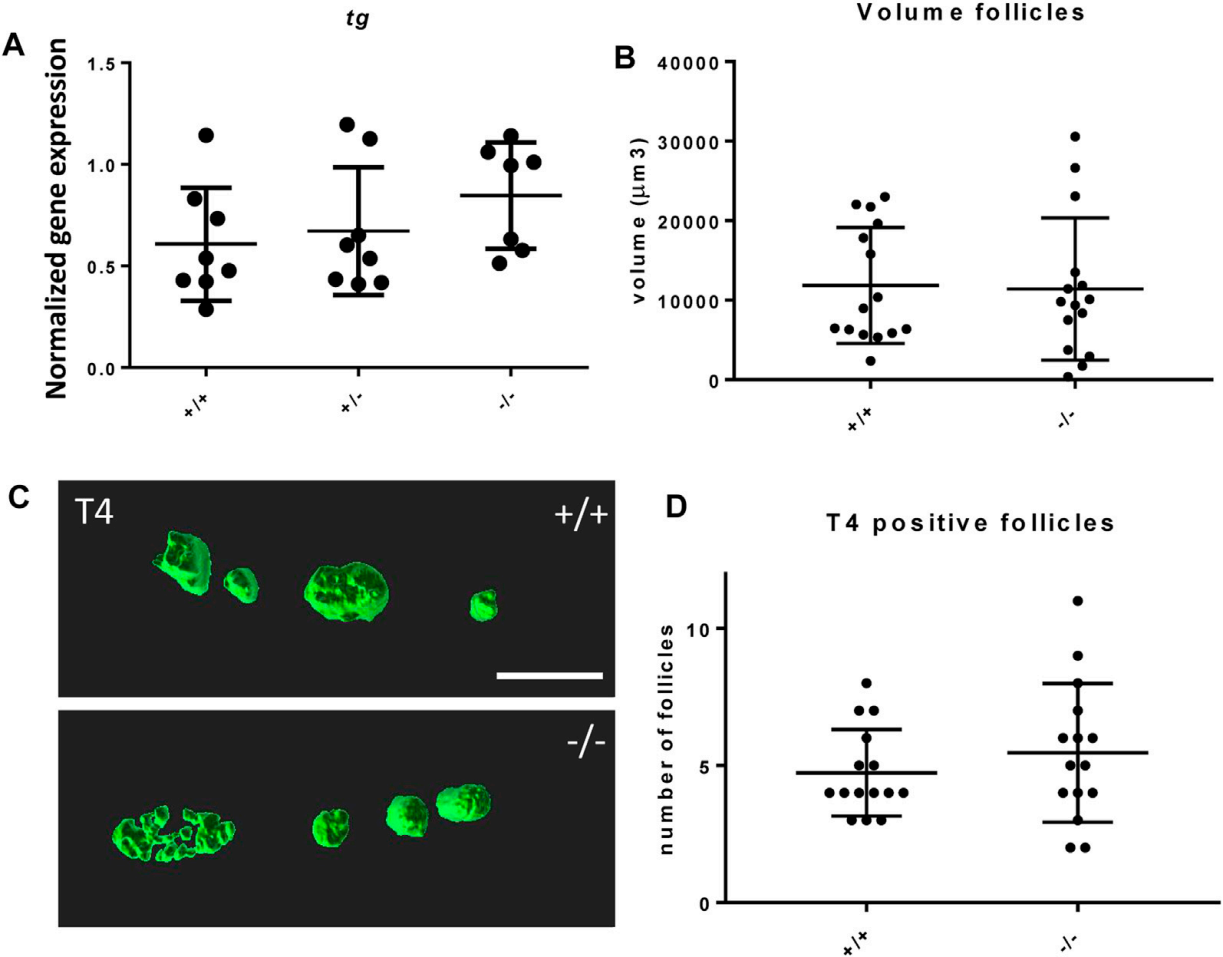
Thyroid follicle development in *foxe1* mutants. **(A)** Normalized gene-expression of thyroid marker thyroglobulin during early development (3 dpf). **(B, D)** Analysis of T4 positive follicles by whole mount immunohistochemistry in 6 dpf larvae showed no difference in mean follicle count or volume. **(C)** Representative images of wild type and mutant thyroid follicles. Larvae were imaged from the ventral side and T4-positive follicle surfaces were rendered from z-stack images using Imaris 9.0 as previously described (Zhang et al., 2021). Scale bar 200 µm. **(D)** Data were assessed for normality with the D’Agostino-Pearson normality test. Normally distributed data were analyzed for statistical differences using a one-way ANOVA and *post hoc* Tukey test or unpaired *t*-test. Non-parametric data were compared with a Kruskal–Wallis test with *post hoc* Dunn’s Multiple comparison test or Mann-Whitney test. Error bars indicate standard deviation.

The effect of the mutation on the development of the thyroid follicles was assessed by gene expression of thyroglobulin (*tg)* and morphological analysis of the thyroid follicles at 6 dpf. The *foxe1* mutants showed no statistically significant gene expression differences in *tg* at 3 dpf compared to wild types (Figure 6A). *tg* expression reflects the function of thyroid follicles (De Felice et al., 1998). Follicle morphology at 6 dpf by T4 antibody staining showed that there were no differences in average number nor size (by volume) of differentiated follicles per individual between mutants and wild types (Figures 6B–D).

### Bone and cartilage formation are adversely affected in the *foxe1* mutants

**FIGURE 7:**
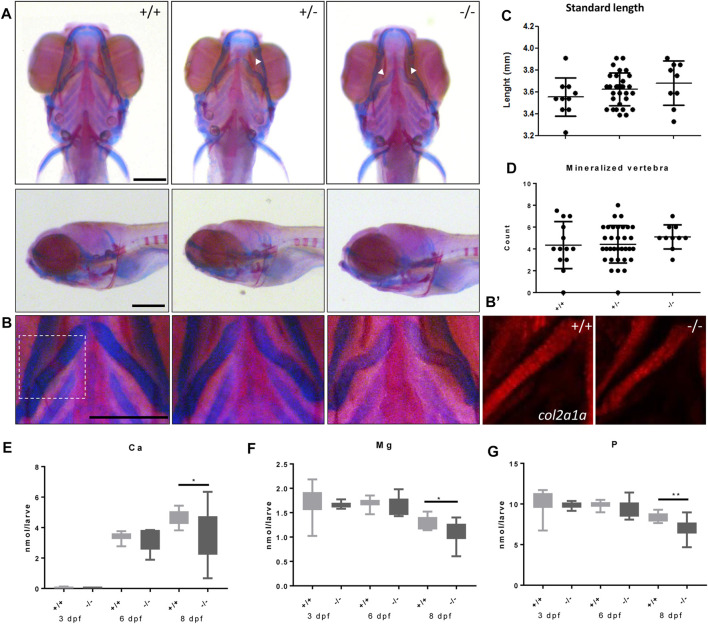
Malformed ceratohyal cartilage and reduced content of Ca, Mg and P in *foxe1* mutants. **(A)** Representative images of wild types and *foxe1* heterozygous- and homozygous mutants, ventral and lateral view. **(B)** High magnification images of ceratohyal phenotype. **(B′)** *Col2a1a* positive cells in the in ceratohyal. **(C)** Standard length at 8 dpf. **(D)** Count of mineralized vertebrae in *foxe1* mutants *versus* wild types at 8 dpf. **(E–G)** Molar calcium, magnesium and phosphorus content in *foxe1* mutants *versus* wild type larvae during early skeletal development three to 8 dpf. *n* = 10–33. Scale bar 200 µm. Data were assessed for normality with the D’Agostino-Pearson normality test. Normally distributed data were analyzed for statistical differences using a one-way ANOVA and post-hoc Tukey test or unpaired *t*-test. Non-parametric data were compared with a Kruskal–Wallis test with post-hoc Dunn’s Multiple comparison test or Mann-Whitney test. Error bars indicate standard deviation.

We next studied the effects of the *foxe1* mutation on craniofacial development. In 19% and 36% percent of heterozygote and homozygote mutants, respectively, a kink in one or both ceratohyal cartilages was observed upon bone and cartilage staining at 8 dpf (Figures 7A, B). We observed this specific collapse after staining, but not during live-imaging studies of 5 dpf zebrafish larvae carrying the *foxe1* mutation homozygously in a transgenic *col2a1a:mCherry* background (Figure 7B’). The morphology of other bone and cartilage structures in the larval head was unaffected. Measurements of head skeletal structures showed no differences between mutants and wild types (Supplementary Figure S3). The larvae’s total body length and the number of mineralized vertebrae at 8 dpf were unaffected (Figures 7C, D). An analysis of the total molar content of essential elements revealed a reduction in the levels of calcium, magnesium, and phosphorus in the *foxe1* mutants at 8 dpf, indicating a more fragile bone structure in mutants (Figures 7E–G).

**FIGURE 8:**
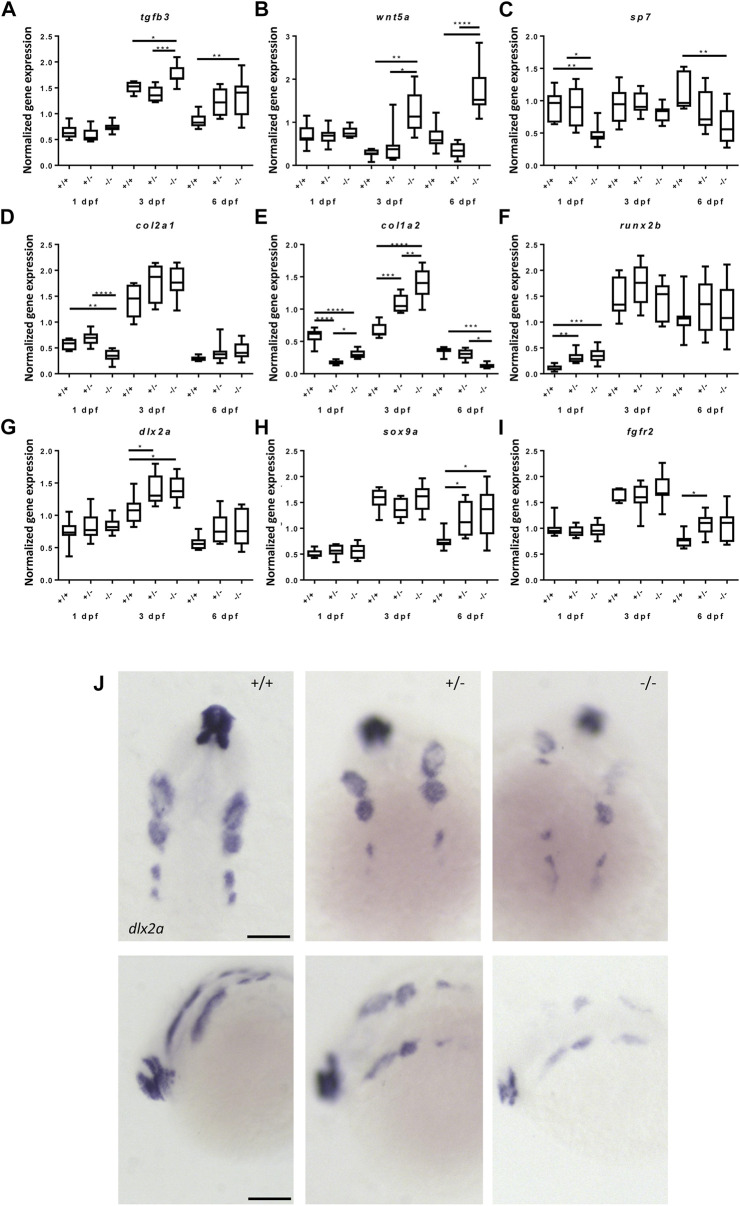
Gene expression of cartilage and bone (precursor) cells is affected in *foxe1* mutants. **(A-I)** Relative gene expression of *tgfβ3, wnt5a, sp7, col2a1, col1a2, runx2b, dlx2a, sox9a and fgfr2* in mutants *versus* wild types. *Axis* description from **(A)** applies to all graphs. **(J)** *dlx2a* positive post migratory neural crest cells at 24 hpf (Prim-5) in wild types, 20% of the heterozygous- and 60% of the homozygous mutants. Asterisks indicate the level of significance: * = *p*< 0.05, ** = *p*< 0.01, *** = *p*< 0.001, **** = *p*< 0.0001 (*n* = 10–15). Data were assessed for normality with the D’Agostino-Pearson normality test. Normally distributed data were analyzed using a one-way ANOVA and post-hoc Tukey test. Non-parametric data were compared with a Kruskal–Wallis test with post-hoc Dunn’s Multiple comparison test. Error bars indicate standard deviation. Scale bar 100 µm.

To better understand the effects of the *foxe1* mutation on head skeleton development, the relative expression of direct target genes of Foxe1 was determined. *Tgfb3* was consistently upregulated in mutants during embryonic and larval development, suggesting a function for Foxe1 as a transcriptional repressor of this gene, as it is for some other genes (Figure 8A) (Perrone et al., 2000; Venza et al., 2011; Lane et al., 2014). *Wtn5a,* reported to be regulated by Foxe1, was also consistently upregulated in mutants (Figure 8B).

Because we observed apparent abnormalities in mineralization in the mutants, we assessed the gene expression of important markers of cartilage and bone development in mutants and wild types. Gene expression levels of *sp7*, *col2a1* and *col1a2* were downregulated at 1 dpf while *runx2b* was upregulated in *foxe1* mutants (Figures 8C–F). At 3 dpf, *col1a2* and *dlx2a* were upregulated in mutants, suggesting that both bone and cartilage development are (indirectly) affected by Foxe1 (Figures 8D,G). S*ox9a* was upregulated at 6 dpf, when it is expressed in both cartilage cells and developing osteoblasts in *foxe1* mutant zebrafish (Figure 8H) (Eames et al., 2012). *Col1a2* was differentially expressed in mutants over time but opposing effects on transcription of *col1a2* were observed at three and 6 dpf (Figure 8E). During the latter timepoint, *col1a2* is specifically expressed in the ceratohyal perichondrium (Eames et al., 2012). The osteoblast-specific transcription factor *sp7* (*Osterix*) was significantly downregulated throughout development of *foxe1* mutant zebrafish. We hypothesize that this is causally associated with the reduced amount of Ca, P, and Mg in mutants, as *sp7* functions as a major regulator of bone development. Indeed, mineralization deficiencies were previously found in *sp7* mutants (Kague et al., 2016; Niu et al., 2017; Chen et al., 2019).

Craniofacial malformations often originate from disrupted regulation of the CNCCs. To investigate whether the *foxe1* mutation had effects on the CNCC cell populations, the spatiotemporal and quantitative expression of the CNCC marker *dl2xa* was assessed. *Dlx2a* relative gene expression by qPCR did not differ significantly between mutants and wild types at 24 hpf (Figure 8G). However, *in situ* hybridization showed that *dlx2a* positive CNCCs populations were affected in 20% of the heterozygous mutants and 60% of the homozygous mutants, this was not observed in wild types (Figure 8J). The various streams of CNCCs showed depletion of *dlx2a*-positive cells and appeared asymmetrically organized or populations were even entirely absent.

## Discussion

The function of FOXE1 in most tissues is as yet not fully understood. Our study shows that *foxe1* is involved in chondrogenesis and osteogenesis in early developmental stages. For the first time we show the localization of Foxe1 protein in developing zebrafish and demonstrate that it is widely expressed; specifically, Foxe1 was expressed in the eyes, the thyroid follicles, the brain, the oral epithelium, the notochordal sheath, and the fin tips. Staining intensity in *in situ* hybridization images did not equal signal strength upon antibody staining in our study. Previously, two reports described the spatiotemporal distribution of *foxe1* transcripts from 11 to 96 hpf in the notochord, the thyroid primordium and the pharyngeal skeleton and oral epithelium (Nakada et al., 2009; Lidral et al., 2015). Nakada et al. reported Foxe1 transcripts in multiple adult zebrafish tissues, most strongly in the eyes, the brain, the gills, and the heart. In human embryos, FOXE1 was detected in the oropharyngeal epithelium and thymus, while proteomic expression data (www.ebi.ac.uk) suggest that it is much more widely expressed in adults (Trueba et al., 2005; Wang et al., 2019). In mice, expression of FOXE1 was specifically found in the thyroid, pharyngeal wall and arches, and in the tongue, the palate, the epiglottis, the pharynx, the esophagus, and the whiskers (Dathan et al., 2002). Together, these data indicate that the FOXE1 expression patterns are evolutionarily conserved across vertebrate species and ontogeny.

To better understand the function of FOXE1, we generated and characterized a zebrafish *foxe1* mutant. We did not obtain frame-shift mutants, which may indicate these were early lethal. It is noteworthy that, as opposed to the previously used mouse and zebrafish models, Bamforth–Lazarus syndrome mutations typically impair FHD DNA-binding, but do not affect the C-terminal protein regions (Castanet and Polak 2010). Therefore, the previously described mouse knockout model may also not be fully representative for the clinical syndrome (De Felice et al., 1998). As a strategy to compromise Foxe1 function, we disrupted the N-terminal NLS sequence of *foxe1*. The N-terminal NLS is involved in nuclear retention while the C-terminal NLS is important for nuclear translocation (Romanelli et al., 2003). We observed a compromised nuclear localization and differential expression of genes downstream of Foxe1 in the NLS-disrupted mutants. Deregulation of NLS-dependent protein import into the nucleus has been linked to cancer and several developmental disorders (McLane and Corbett 2009). For instance, changes in the NLS sequence of the SRY gene cause Swyer syndrome because of reduced nuclear translocation of the protein (Harley et al., 2003).

Strict regulation of FOXE1 activity appears essential for normal development, as dysregulation has detrimental effects. In humans, two homozygous FOXE1 probands have been described with the mutations S57 N and A65 V (Clifton-Bligh et al., 1998; Castanet et al., 2002). These mutations in highly conserved FHD amino acids impair FOXE1 DNA binding capacity partly and completely, respectively (Castanet et al., 2002). Based on the phenotypes in these individuals, features of the Bamforth–Lazarus syndrome were deemed more extensive, as the capacity of FOXE1 DNA binding decreased (Castanet et al., 2002). However, a homozygous gain-of-function mutation (R73 S), which increased DNA binding capacity, led to the same Bamforth–Lazarus phenotypes as loss-of-function variants (Carre et al., 2014). Similar to these loss- and gain-of-function mutations, we show that in our zebrafish model the transcriptional activity of Foxe1 is compromised, in our case due to disrupted nuclear localization.

A typical symptom of Bamforth-Lazarus syndrome is cleft palate, which refers to a defective fusion of the two palatal shelves (Castanet and Polak 2010; Sarma et al., 2022). The ethmoid plate of the zebrafish is analogous to the human hard palate, and cleft phenotypes have been described in multiple zebrafish mutants (Mork and Crump 2015). These phenotypes include rough anterior edges of the ethmoid plate at varying degrees of severity, or a lack of cells in the medial ethmoid plate (Swartz et al., 2011; Mork and Crump 2015; Duncan et al., 2017). Although we did not observe obvious effects of the *foxe1* mutation on the morphology of the ethmoid plate, we did find differential expression of *sox9, tgfβ3* and *col2a1.* These are important factors for the development of the ethmoid plate, thus more subtle defects in chondrogenesis of this structure cannot be ruled out.

In our *foxe1* mutant, we observed a kink of the ceratohyal cartilage, reminiscent of previously reported kinks in the ceratohyals of *lrp5* mutants (also after bone and cartilage staining), which aimed to mimic osteoporotic phenotypes (Bek et al., 2021). This structure is the only head skeleton cartilage that mineralizes *via* endochondral ossification at this timepoint (Tonelli et al., 2020). As the kink is observed at the middle of the ceratohyal, which is the primary ossification center, a decreased ossification may have led to a collapse of the structure after the staining procedure which expands soft tissues slightly. Nakada et al. showed specific upregulation of *fgfr2* surrounding the ceratohyal in Foxe1 morphants, which is in line with the increased *fgfr2* transcripts levels we observed in our mutants (Nakada et al., 2009). *FGFR2* gain- and loss-of-function mutations have been associated with multiple skeletal syndromes such as bent bone dysplasia, in which long bones are bent and contain smaller hypertrophic chondrocytes in the growth plate and a thickened periosteum (Merrill et al., 2012). It is thus tempting to speculate that the *foxe1* mutation has led to a less rigid structure of the ceratohyal bone which led to a collapse upon staining.

It was proposed by Xu and others (2018) that a Fox code for craniofacial patterning may exist. Fox-F and Fox-C-proteins were found to be individually redundant for cartilage morphogenesis in zebrafish. However, there were severe additive effects in case of multiple Fox-mutations. Xu and others described reduced or absent lower jaw cartilage elements caused by Fox-F mutations and malformed upper facial cartilages associated with Fox-C mutations (Xu et al., 2018). Interestingly, this study reported specifically that the ceratohyal cartilages were not affected by any of the mutations. Together with our data, this indicates there may be a specific role for Foxe1 in ceratohyal patterning within the suggested Fox code (Xu et al., 2018).

Another clinical symptom reported in two infants with Bamforth–Lazarus syndrome, is delayed growth. This was specified in one individual as a severe delay in bone maturation with absent ossification centers in the tibial epiphyses (Castanet et al., 2002). Our model may have subtly recapitulated this clinical symptom associated with a *FOXE1* mutation at 8 dpf in the ceratohyals.

We further observed a reduced Ca, Mg and P content in mutant larvae at 8 dpf compared to wild type siblings. This is in line with the reduced rigidity of the ceratohyals and with the observed downregulation of *sp7*. S*p7* functions as a master gene for osteoblast differentiation from *sox9* and *runx2* positive progenitors (Akiyama et al., 2005; Flores et al., 2006)*.* Sp7^−/−^ mice develop cartilage normally, but no osteoblasts or bone are formed, while the mutation is embryonically lethal (Nakashima et al., 2002; Sinha and Zhou 2013). Cartilage formation was also normal in zebrafish with truncated *sp7*, but bone growth and mineralization defects were observed as well as the absence of teeth (Kague et al., 2016; Niu et al., 2017; Chen et al., 2019). Altered expression of other Fox-family proteins in CNCCs inhibited bone development and expansion of cartilages, which may be reflected in the upregulation of chondrocyte markers and *sp7* downregulation in our mutants (Xu et al., 2018). *Col1a2* was also downregulated in our mutants at 6 dpf. Mutation of this gene causes osteogenesis imperfecta, characterized by low bone mass and high bone fragility, also previously modeled in zebrafish (Gistelinck et al., 2016).

An essential process in CNCCs is EMT, in which morphological and behavioral characteristics change to promote migration (Theveneau and Mayor 2012). Loss- and gain-of-function studies of FOXE1 in thyroid cancer cells show that FOXE1 may be an EMT modulator (Morillo-Bernal, Fernandez, and Santisteban 2020). Previously, Nakada and others suggested that CNCC migration and specification following EMT is not affected by *foxe1* knock-down. In our study, we observed no differences in total expression of *dlx2a*; however, the spatial distribution of *dlx2a* at 24 hpf was severely altered in a subset of heterozygous and homozygous mutants. These differences may be mediated through *tgfb3*, which was consistently upregulated in our mutants, likely due to dysregulation of Foxe1. At 24 hpf, *tgfb3* is expressed in post-migratory CNCCs in the pharyngeal arches (Perrone et al., 2000; Cheah et al., 2010; Venza et al., 2011). Both overexpression and knock-down of *tgfb3* resulted in reduced amounts of post-migratory *dlx2a*-positive CNCCs in a study by Cheah and others with similar distorted stream morphology to *foxe1* mutants. Despite the distorted *dlx2a* expression in CNCCs, the craniofacial cartilages developed normally, as was the case for most cartilage structures in our *foxe1* mutants (Cheah et al., 2010). Studies on knockdown and knockout of dlx2a in zebrafish give conflicting evidence on the importance of *dlx2a* on CNCC survival and craniofacial malformations. Knockdown resulted in increased apoptotic CNCC death and lead to defects in the ceratobranchials (Sperber et al., 2008). However, stable *dlx2a*−/− mutants showed no apparent malformations up to adulthood (Yu et al., 2021). Although the effects of disrupted *dlx2a* expression are unclear, our findings strongly suggest that Foxe1 is involved in neural crest cell processes.

In the present study we found consistent Foxe1 expression and colocalization with T4 in the developing thyroid follicles of zebrafish. This suggests a role for this transcription factor in thyroidogenesis in zebrafish, although this was previously debated (Nakada et al., 2009). However, Lidral and others characterized the expression of the zebrafish *foxe1* enhancer region hsCNE-67.7 and reported that it also colocalizes with T4 at 96 hpf, which supports our findings (Lidral et al., 2015). Although the presence of Foxe1 in the developing follicles suggests a developmental function, its exact role requires further study*.* In regard of Bamforth–Lazarus characteristics, there is clearly a role for the FOXE1 FHD in thyroid development, but the C-terminal PAS region of the protein is also important. Shorter PAS length in humans is associated with susceptibility to thyroid dysgenesis. However, it does not affect the binding of FOXE1 to the promoters of the *TGFβ3* and *MSX1* genes, which are important for skeletal development (Carre et al., 2007; Venza et al., 2011; Pimentel et al., 2017). The absence of the PAS region in the zebrafish Foxe1 protein may partially explain why, up to now, no function of Foxe1 in thyroid development was found.

Taken together, this study shows that the developmental functions of Foxe1 are at least partly conserved between humans and zebrafish. We reported widespread expression of *foxe1* during development and showed that small amino acid changes in the NLS alters its function in craniofacial bone development. We describe novel factors associated with Foxe1 that guide CNCC development and bone formation. Our model provides critical new insights into how *foxe1* mutations affect craniofacial development and may aid in elucidating FOXE1-related disease etiologies, such as in Bamforth-Lazarus syndrome. Our observations that only in a subset of the *foxe1* mutants a phenotype is obvious, indicates that a set of unknown additional factors determines whether or not an aberrant phenotype develops. Gene-gene and gene-environment interactions have been broadly reported to play a significant role in craniofacial malformations in general (Raterman et al., 2020). Some of these interactions have also been specifically associated with the SHH pathway, and our future studies will focus on these interactions and the persistence of skeletal defects in adults. The fact that the *foxe1* gene in our model system is compromised but not completely abolished as it would be in a knockout, makes this a powerful model system to separate the relative influence of genetic and environmental risk factors.

### Methods

#### Animals

Animal procedures were carried out in accordance with the Dutch Animals Act and European laws. Ethical approval for the experiments was granted by Radboud University’s Institutional Animal Care and Use Committee (AVD10300202115245). Zebrafish (*Danio rerio*) were raised and kept under standard husbandry conditions (28 °C under a 14 h light/10 h dark cycle) in the Radboud University Zebrafish Facility. For breeding, males and females were separated overnight by a transparent divider in breeding tanks. The next day, at the start of the light period, the divider was removed and breeding water was added to induce spawning. Eggs were collected and transferred to Petri dishes with E3 medium (5 mM NaCl, 0.17 mM KCl, 0.33 mM CaCl_2_, 0.33 mM MgSO_4_, 0.00001% Methylene Blue). Larvae were raised in an incubator at 28.5 °C with a 14 h light/10 h dark cycle and medium was refreshed daily.

### CRISPR/Cas9 mutant generation

Using CHOPCHOP (https://chopchop.cbu.uib.no) an efficient single guide (sg) RNA was selected at the 5′ end of the target exon. sgRNAs were generated using templates for *in vitro* transcription as previously described (Schellens et al., 2021). Briefly, a 20 nt (GGC​CGC​AAA​GAG​GCC​GTC​GG) target specific oligonucleotide with a T7 promotor sequence (5′-TAA​TAC​GAC​TCA​CTA​TA-3′) and a complementary region (5′-GTT​TTA​GAG​CTA​GAA​ATA​GCA​AG-3′) to a constant oligonucleotide encoding the reverse complement of the tracrRNA tail were annealed. Using Phusion™ High-Fidelity DNA Polymerase (New England Biolabs, Ipswich, MA, United States #M0530 L) the ssDNA overhang was filled. Next, the template was purified using the GenElute™ PCR cleanup kit. *In-vitro* transcription was performed using T7 MEGAshortscript kit. The transcripts were purified using MEGAclear transcription clean up kit.

Before injection, the sgRNA/Cas9 ribonucleoproteincomplex was formed by incubating a mix of 80 ng/μL sgRNA, 800 ng/μL Cas9 protein (Intergrated DNA Technologies), 0.2 M KCl and 0.05% phenol red at 37 °C for 5 min. Injection needles (World Precision Instruments, Friedberg, Germany, #TW120F-3) were prepared using a micropipette puller (Sutter Instrument Company, Novato, CA, United States, Model P-97). Wild type AB strain zebrafish embryos were collected and injected at the single cell stage with 1 nL sgRNA/Cas9 mixture using a Pneumatic PicoPump pv280 (World Precision Instruments, Friedberg, Germany). Subsequently, embryos were raised at 28.5 °C in E3 medium. At 24 hpf, a sample of the injected embryos (8 pools of three embryos) was analyzed for the presence of indels. Therefore, genomic DNA was extracted from injected and control embryos. The target site was amplified in a PCR reaction (fw, 5′-CCC​TCT​GAA​ACC​ACT​CTT​CCA​G-3’; rev, 5′-TGT​ATG​GTG​GTT​TGC​CTC​GC-3′) and products were analyzed for indel induction by HRM as described previously (Thomas et al., 2014). Sanger sequencing was used for confirmation of indels. In the F1 generation, one indel was selected and outcrossed. After outcrossing with wild types the F3 generation was used for experiments.

### Immunohistochemistry

Immunohistochemistry was performed as described earlier (Schellens et al., 2021). Larvae were euthanized and fixated in 4% PFA overnight and embedded in Optimal Cutting Temperature (Agar Scientific) for cryo-sectioning. 7 µm horizontal sections were mounted on a glass slide and washed in PBS before incubation in PBS with 0.01% Tween (Sigma) for 20 min. After PBS rinse, blocking was performed using 10% NGS (Thermo Fisher) 2% BSA (Sigma) before overnight primary antibody incubation in blocking buffer at 4 °C. The polyclonal antibody for zebrafish Foxe1 was custom-made at Boster Bio (DZ41149, using C-terminal peptide AVGSSGAYIRHPAYSG as the immunogen) and it was applied at a dilution of 1:500. The T4 antibody (MP Biomedicals rabbit anti-T4) was diluted to 1:1,000. Secondary antibodies (Alexa Fluor 488/568 Thermo Fisher) were applied at 1:800 in blocking buffer. After washing, a short incubation with 300 nM DAPI was performed before post-fixation in 4% PFA. Sections were then sealed in FluorSave (Merck Millipore). Signals were imaged using an SP8x confocal laser scanning microscope (Leica-microsystems) with Leica Application Suite X software at magnifications x20 or 100x.

Whole mount immunohistochemistry was performed exactly as described previously (Hammond-Weinberger and ZeRuth 2020) using T4 antibody (MP Biomedicals rabbit anti-T4) diluted at 1:1,000 for whole embryos and Foxe1 Boster Bio (DZ41149) at 1:500 on scales. Z-stack images of the T4 staining were taken on an SPX8 confocal microscope at magnification ×20 and were processed by surface rendering using Imaris 9.0 software to calculate volumes as previously described (Zhang et al., 2021).

### *In situ* hybridization

Whole mount *in situ* hybridization was performed as previously reported (Thisse and Thisse 2008) (Gebuijs et al., 2019). Probes were generated using respective primers: *dlx2a* fw, 5′-AGT​GTG​CTT​TTG​CGG​TAT​GA-3′, rev, 5′-AAT​ATG​GTC​CCG​GCG​CTA​AC-3′ and *foxe1* fw, 5′-ATG​CCT​GTG​GTT​AAA​GTG​GAG​AGT-3′, rev, 5′-TGG​CCC​ATA​ATG​CTG​AGT​GCT-3’. In brief, sequences were amplified by PCR and cloned in pGEM^®^-T Vector (Promega). Sanger sequencing was used to confirm positive clones. Plasmid linearization was performed using Notl and Ncol (Thermo Fisher Scientific), before purification with GeneJET PCR purification kit (Thermo Fisher Scientific). Digoxigenin (DIG) labeling of the probe was performed using the DIG labeling Sp6/T7 Kit (Roche). Samples were fixed in 4% PFA for 12 h. Digestion with Proteinase K (Sigma-Aldrich) was applied to samples >2 dpf for 30 min at RT. Samples were pre-hybridized at 70°C for 2 h in hybridization mix (HM) (50% formamide (VWR), five x saline-sodium citrate buffer (SSC), 50 μg/ml heparin (Sigma-Aldrich), 500 μg/ml tRNA (Sigma-Aldrich), 0.1% Tween20 and 9.2 mM citric acid). Then, hybridization with DIG-labeled anti-sense probes was performed overnight at 70°C in HM (∼100 ng/ml probe). After hybridization, samples were washed in gradients of HM (without tRNA and heparin) and SSC to PBT and then samples were pre-incubated with 2% NCS (HyClone) in PBT. Next, samples were incubated with anti-DIG AP fragments (Roche) (1:2000) in 2% NCS overnight at 4°C. After washes, labeling was performed using Nitro Blue Tetrazolium (NBT)/5-Bromo-4-chloro-3-indolyl phosphate (BCIP) (Roche) in alkaline phosphatase buffer (100 mM Tris (pH 9.5), 50 mM MgCl_2_, 100 mM NaCl and 0.1% Tween 20). For imaging, larvae were cleared in a 2:1 mixture of benzyl benzoate (Merck) and benzyl alcohol (Merck). Images were acquired with a binocular microscope (Leica DMRE) using Leica Application Suite (LAS 3.3, Leica).

### Quantitative polymerase chain reaction

Total larval RNA was extracted in 400 μL Trizol reagent (Invitrogen, Carlsbad, United States of America) following the manufacturer’s protocol and quantified using a NANODROP 1000 spectrophotometer. For cDNA synthesis, the iScript cDNA Synthesis Kit (Bio-Rad, Cressier, Switzerland) was applied. Used primers are listed in Table 1. The qPCR mix contained 10 μL SYBR green mix (2X) (Bio-Rad, Hercules, United States of America) and qPCR was performed using a CFX 96 (Bio-Rad) machine. Relative expression was calculated based on normalization against two reference genes: elongation factor alpha (*elf1a*) and ribosomal protein L13 (*rpl13*) (Vandesompele et al., 2002).

**TABLE 1:** Primer sequences of qPCR experiments.

Target	Forward primer	Reverse primer
*col1a2*	GCG​ACT​TTC​ACC​CCT​TAG​GA	TGC​ATA​CTG​CTG​GCC​ATC​TT
*dlx2a*	GAC​TCA​GTA​TCT​GGC​CTT​GC	CTG​CTC​GGG​TGG​GAT​CTC​T
*msx1a*	CTC​CCG​TTT​AGC​GTT​GAA​GC	GTG​TTT​TCT​CAG​AGG​GCA​CG
*runx2b*	GGG​CCA​AAC​GCA​GAT​TAC​AG	TCT​GTC​GAA​CCT​GGA​AGA​CG
*sp7*	GGA​TAC​GCC​GCT​GGG​TCT​A	TCC​TGA​CAA​TTC​GGG​CAA​TC
*sox9a*	GCC​ATC​TTC​AAA​GCG​CTC​CA	GTT​TCA​GAT​CCG​CTT​TGC​CTG
*tg*	CGC​CAT​TTA​GTC​TCC​GCT​CT	TCC​ACG​TAC​ACA​GAG​GCA​AC
*tgfβ3*	GGA​CCG​AGC​AGA​GAA​TCG​AG	CGT​CGA​AGG​AAA​CCC​ACT​CA
*wnt5a*	ACG​CAA​ACT​CAT​GGT​GGT​CT	GCC​CCT​TCT​CCG​ATG​TAC​TG
*rpl13*	TCT​GGA​GGA​CTG​TAA​GAG​GTA​TGC	AGA​CGC​ACA​ATC​TTG​AGA​GCA​G
*elf1a*	GGGCAAGGGCTCCTTCAA	CGCTCGGCCTTCAGTTTG

### Bone and cartilage staining

Staining was performed as described by Walker and Kimmel with small modifications (Walker and Kimmel 2007). Larvae were euthanized on ice and fixed for 1 h in 2% PFA. After washing with 100 mM Tris/40 mM MgCl_2 (_pH 7.5) larvae were incubated in 0.04% Alcian Blue (Sigma-Aldrich)/40 mM MgCl_2_ for 2.5 h. After gradual rehydration, bleaching was performed using 3% H_2_O_2_/0.5% KOH. After two short incubations in 25% glycerol/0.1% KOH bone staining with 0.03% Alizarin Red (pH 7.5, Sigma-Aldrich) for 1 h. Finally, larvae were imaged in 3% methylcellulose using Leica Application Suite (LAS 3.3, Leica).

### Live imaging

Wild type zebrafish and Foxe1 mutants in a transgenic *col2a1a:mCherry* background were anaesthetized at 5 dpf and embedded in agarose before short term live imaging on an SPX8 confocal microscope at magnification ×20.

### Inductively Coupled Plasma - Optical Emission Spectroscopy

Larvae were euthanized on ice and rinsed in ultrapure water, transferred to 2 ml Eppendorf tubes and any excess liquid was removed. 100 µL of 65% nitric acid (HNO_3_) was added and the next day, the nitric acid containing the dissolved samples was added to 6 ml ultrapure water. Inductively Coupled Plasma - Optical Emission Spectroscopy (ICP-OES) was used to determine the molar contents of calcium, phosphorus, and magnesium in the samples. Samples were measured on an ARCOSMV (Spectro, Kleve, Germany) on axial view, with 1400 W plasma power. Samples were nebulized with a seaspray nebulizer combined with a cyclone chamber and an argon flow of 0.7 L/min.

## Data Availability

The original contributions presented in the study are included in the article/Supplementary Files, further inquiries can be directed to the corresponding author.

## Abbreviations

BSA; Bovine serum albumin, CNCC; Cranial neural crest cells, COL1A2; Collagen type I alpha 2 chain, COL2A1; Collagen type II alpha 1 chain, CP/CLP; Cleft palate/Cleft lip and palate, CRISPR; Clustered Regularly Interspaced Short Palindromic Repeats, DAPI; 4′,6-diamidino-2-phenylindole, DPF; Days post fertilization, EMT; Epithelial mesenchymal transition, FGFR2; Fibroblast growth factor receptor 2, FHD; Forkhead domain, FOXE1; Forkhead transcription factor E1/TTF-2, GLI2; GLI Family Zinc Finger 2, GWAS; Genome wide association study, HPF; Hours post fertilization, HRM; High resolution meltcurve, LPR5; Low-density lipoprotein receptor-related protein 5, MPF; Months post fertilization, MSX1; Msh Homeobox 1, NGS; Normal goat serum, NIS; Sodium/iodide cotransporter, NLS; Nuclear localization signal, PAS; Polyalanine stretch, RUNX2B; RUNX family transcription factor 2 b, SHH; Sonic hedgehog, SOX9A; SRY-box transcription factor 9a, SP7; Sp7 Transcription Factor, T4; Thyroid hormone, TG; Thyroglobulin, TGFB3; Transforming Growth Factor Beta 3, TPO; Thyroid peroxidase, TTF-2; Thyroid transcription factor 2/FOXE1, WNT5A; Wnt Family Member 5 A.
