# Supplementary material for: Disruption of the foxe1 gene in zebrafish reveals conserved functions in development of the craniofacial skeleton and the thyroid
Source: Front Cell Dev Biol. 2023 Mar 13;11:1143844. doi: 10.3389/fcell.2023.1143844 (PMC10040582; doi:10.3389/fcell.2023.1143844)
Supplement: Supplementary file 1 [file DataSheet1.PDF]

Supplementary Material

Disruption of the foxe1 gene in zebrafish reveals conserved functions in development of the craniofacial skeleton and the thyroid

Sophie T. Raterman[1,2], Johannes W. Von den Hoff[1], Sietske Dijkstra[2], Cheyenne de Vriend[2], Tim te Morsche[2], Sanne Broekman[3], Jan Zethof[2], Erik de Vrieze[3], Frank A.D.T.G. Wagener[1], Juriaan R. Metz[2]

*** Correspondence:** Sophie Raterman: Sophie.Raterman@radboudumc.nl

1 Supplemental methods

Western blot

Larvae were euthanized on ice and total protein was extracted by homogenization in lysis buffer. 10 ug protein was added to SDS sample buffer which was loaded on a 10% Mini-PROTEAN® TGX™ gel (Biorad) before blotting on a nitrocellulose membrane (Biorad). The blot was washed with TBST and blocking was performed using 3% skimmed milk powder. Primary antibodies, acetylated tubulin (Sigma, T6793) and Foxe1 (Boster Bio, DZ41149) were diluted at 1:3000 in 0.5% skimmed milk powder/TBST. Secondary antibody (Anti-rabbit IgG-peroxidase A9169 and Anti-mouse IgG-peroxidase A4416, Sigma Aldrich) incubation lasted 1 hour at (1:80 000). Secondary antibodies were visualized using DAB as chromogen.

Homology modelling

The SWISSPROT model server was used to select a suitable template FOXE1, homology templates were assessed according to an adapted version of the modeling workflow of Waterhouse and colleagues (Waterhouse et al. 2018). Selection criteria included sequence identity, model resolution and GMQE score. After selecting the appropriate homology templates, the respective templates were loaded into the free-downloadable software YASARA (Version 21.6.2) (YASARA Biosciences GmbH) to inspect each amino acid independently. These were aligned and merged to form the in-silico FOXE1 winged helix protein model.

Morphometric analyses

Alizarin red- and Alcian blue-stained zebrafish were imaged and morphometric measurements were performed using ImageJ. Using standardized landmarks, the Meckel's-palatoquadrate angle, the ceratohyal angle, head width at the Meckel's palatoquadrate joint, head width at the interhyal-ceratobranchial interface, the Meckel's-ceratohyal distance and the total head length starting at the notochord were measured.

2 Supplementary Figures

Supplemental figure 1

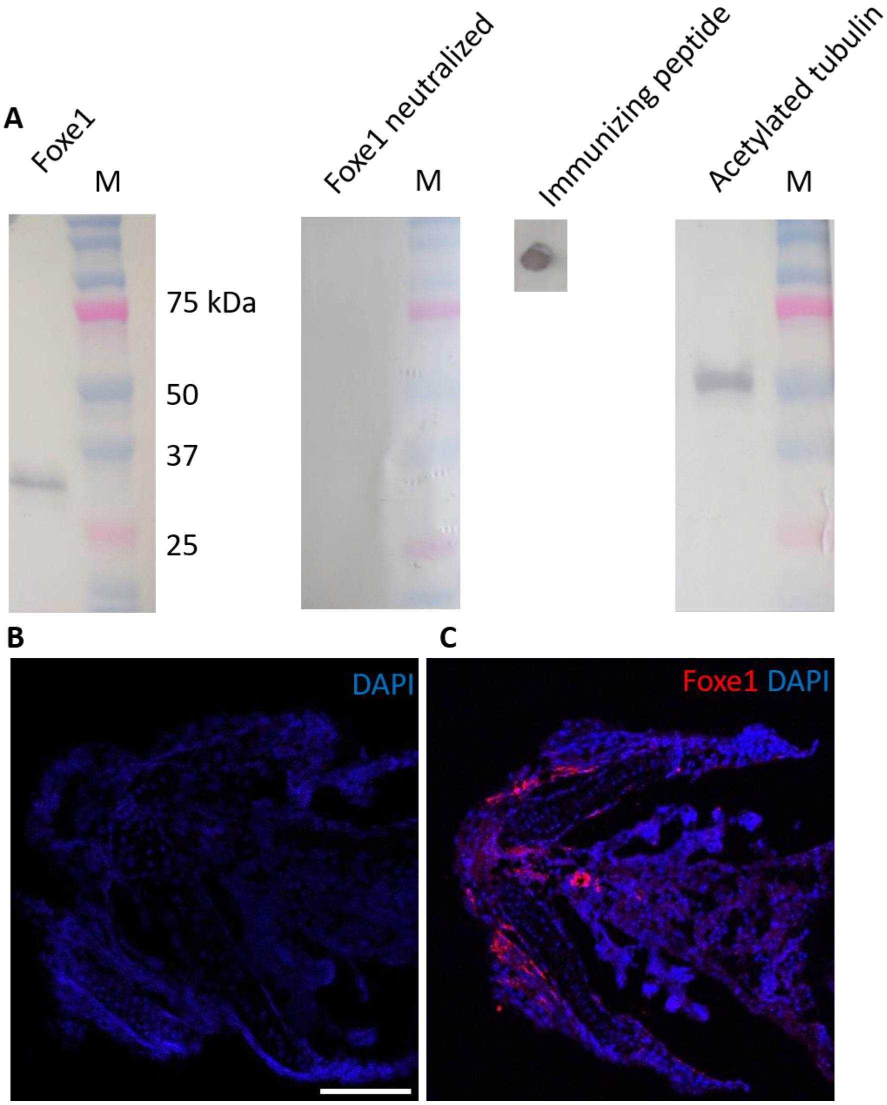

Figure S1. Foxe1 antibody validation. A) Western blot of whole larva homogenates showing a single band of predicted size with the custom-made Foxe1 antibody; when the antibody was neutralized (pre-incubated with 1 µg/ml of the immunizing peptide for 2 h) the signal disappeared, further confirming specificity. A spot blot of the immunizing peptide detected with the Foxe1 antibody is shown in the inset. Acetylated tubulin antibody served as the positive control. B) Immunohistochemistry; no primary antibody control and (C) corresponding sequential section incubated with primary antibody. Scale bar 200 µm.

Supplemental figure 2

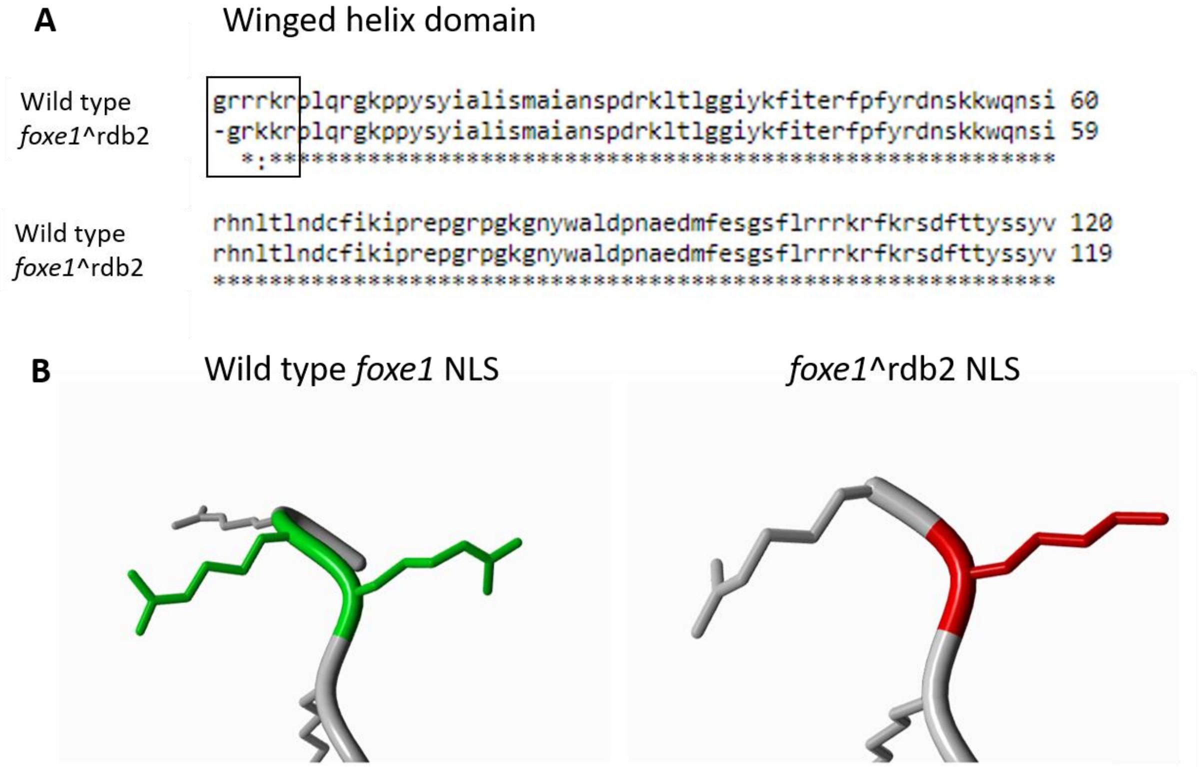

Figure S2. **Homology modeling illustrates structural change in *foxe1*^rdb2 NLS sequence.** A) The sequence of the winged helix domain of zebrafish Foxe1 was modeled through homology modeling using SWISSPROT and YASARA. Subsequently, the mutation was visualized in the 3D structure. B) NLS sequences of the Foxe1 model with and without the mutation are shown here. The wild type nuclear localization sequence contains mostly arginine residues (green). The mutations, R31del and R32K, in the NLS sequence are modeled in red. As shown, the NLS sequence is drastically shortened in the mutant.

Suplemental figure 3

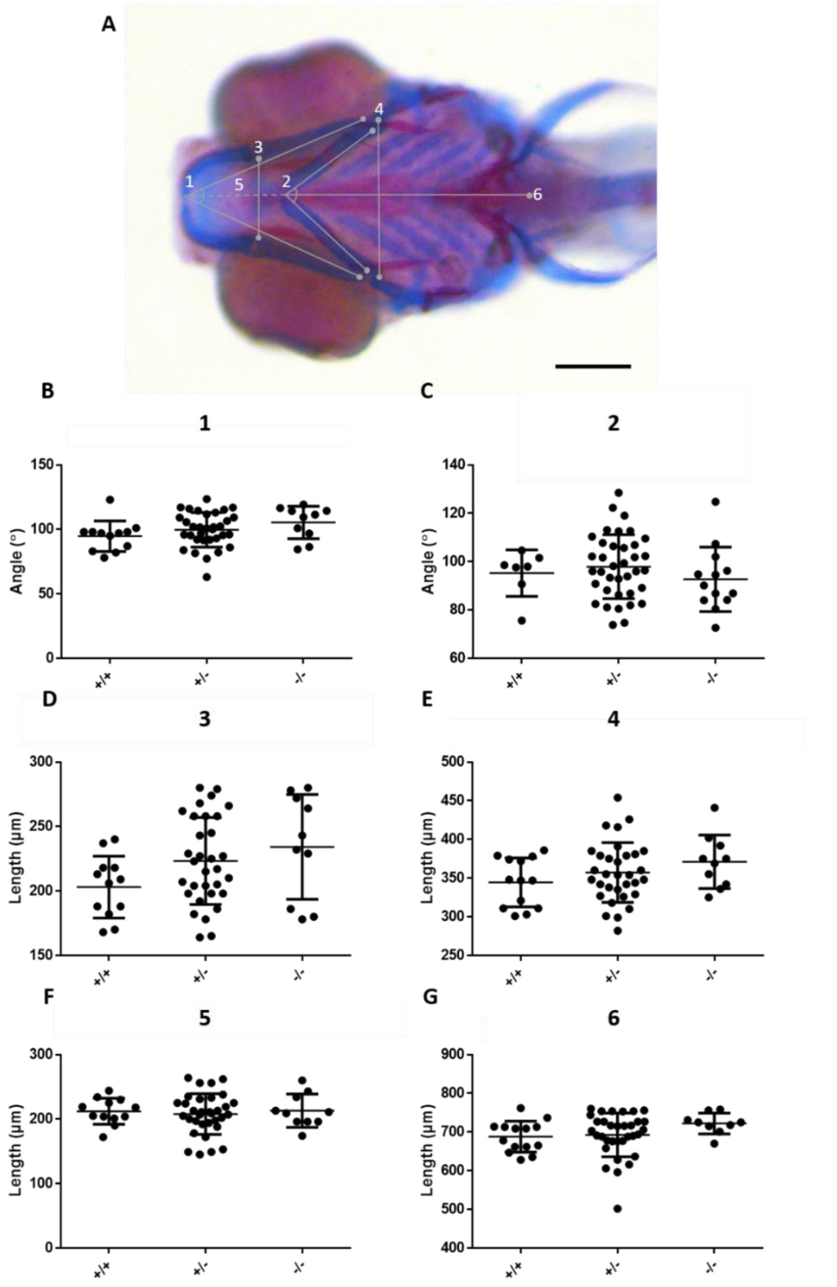

Figure S3. ***Foxe1*** **mutant head skeleton morphometric analyses at 8 dpf show no differences between wild type and mutant fish.** Various measurements from bone and cartilage stained larval 8 dpf heads were taken. A) A representative larval fish imaged from the ventral side with landmarks for craniofacial morphometrics (B-G): (1) the Meckel's-palatoquadrate angle, (2) the ceratohyal angle, (3) head width at the Meckel's palatoquadrate joint, (4) head width at the interhyal-ceratobranchial interface, (5) the Meckel's-ceratohyal distance and the (6) total head length starting at the notochord. n= 11-33. Scale bar 200 µm. Data were assessed for normality with the D'Agostino-Pearson normality test. Normally distributed data were analyzed using a one-way ANOVA and post-hoc

Tukey test. Non-parametric data were compared with a Kruskal-Wallis test with post-hoc Dunn’s Multiple comparison test. Error bars indicate standard deviation.
